# Spatial Cleaning Action of Ultrasonic Irrigation on *Enterococcus faecalis* Biofilm

**DOI:** 10.3390/dj8020042

**Published:** 2020-05-07

**Authors:** Michael C.C. Tse, Gary S.P. Cheung

**Affiliations:** 1 Private Practice, Central, Hong Kong SAR, China; tsemichael@ymail.com; 2 Division of Restorative Dental Sciences, Faculty of Dentistry, University of Hong Kong, Pokfulam, Hong Kong SAR, China

**Keywords:** activated irrigation, ultrasonic, spatial effect, biofilm, endodontic treatment, root canal

## Abstract

This study aimed to examine the spatial cleaning effect of ultrasonic irrigation in simulated root canal with oblong cross section in the absence of antimicrobial agent. A 7-day *E. faecalis* biofilm was cultivated in a rectangular, simulated canal model and subjected to passive ultrasonic irrigation (PUI) with sterile saline for 5 minutes. After that, the biofilm was examined by confocal microscopy after bacterial viability staining at 58 sites around and beyond the endosonic file. Results showed that, at the vicinity of the file, the amount of viable bacteria ranged from 13.1% (2.75 mm from the tip) to 40.5% (4.5 mm level). Lesser amounts of live bacteria were observed within 1 mm from the vibrating file, which amount increased for sites farther away. At 3 mm distance, the amount of bacteria (35.5 to 64.4%) was significantly greater than areas situated close to the vibrating file (*P* < 0.01). Sites next to the anti-nodes of file had less bacteria remaining than sites near the nodes (*P* = 0.050). Apically (0.5 mm or more), the amount of bacteria was significantly greater than that at the file tip (16.4%) (*P* < 0.05). It was concluded that PUI is able to dislodge a single-species biofilm, provided that they are situated in close vicinity to the vibrating file.

## 1. Introduction

An important aim of endodontic treatment is to rid the root canal system from infectious agents. A clinical cohort study has clearly demonstrated the significant role of (cultivable) bacteria present within the root canal before obturation in lowering the chance of success for root canal treatment [1]. A strong association has been found between the presence of intracanal infection and non-healing apical periodontitis after endodontic treatment [2,3,4].

Microorganisms found in infected teeth associated with periapical lesions were shown to organize themselves in a biofilm architecture attaching to the root canal wall [2,5]. Sjögren et al. [1] commented that bacteria are often present in areas of the root canal that cannot be reached by chemo-mechanical debridement. It was alarming that, even with the use of operating microscope and contemporary root canal preparation techniques, microorganisms could still be isolated in 88% of mandibular molar roots after non-surgical endodontic treatment [6]. Indeed, most bacteria were found in recessed areas and diverticula of the root canal system [6], which could become microbial niches that lead to the development of (refractory) apical periodontitis. It has been commented that eradication of all bacteria from the complex root canal system is close to impossible [2].

Many instruments or instrumentation techniques have been proposed for root canal preparation, with some notable advancement in the past two decades. The introduction of nickel-titanium (NiTi) rotary instruments has helped to produce a predictable shape to the root canal [7,8], reduce the amount of preparation errors and increase the rate of successful treatment [9]. Rotary instruments tend to produce round preparations (in cross section) [10,11]. However, root canals seldom are round in cross section before treatment, especially in their middle and coronal two-thirds [12]. The final preparation is either situated in the centre or at one side of root canals with oblong cross section [11,13,14], leaving variable amounts of un-instrumented root canal wall and (probably infected) debris in recessed areas [12,15,16].

Ultrasonic irrigation has been shown to be significantly more effective than needle-and-syringe irrigation in dislodging debris and smear layer [17,18,19,20,21] and, possibly, biofilm [22]. The ultrasonically-activated endodontic file, also known as endosonic file, can also prepare the canal wall due to its dentine cutting ability. However, when the endosonic file is intentionally brought into contact with the canal wall for purpose of ultrasonic cutting of dentine, the cleaning efficacy is reduced in comparison with those endosonic files that are used in a non-cutting manner [23]. This mode of usage, that is, agitation of the root canal irrigant without simultaneous instrumentation is known as *Passive Ultrasonic Irrigation* (PUI) [24]. Upon activation, the endosonic file vibrates in a sinusoidal waveform, with the tip being one of the places along the file that show the greatest amplitude of movement [25]. There are sites along the vibrating file that look stationary; these locations are called the nodes. Those sites showing the greatest vibration amplitude are called anti-nodes [25]. That waveform and amplitude of vibration might be altered, if there should be any contact between the file and the canal wall [26]. A recent systematic review [27] has indicated that the use of ultrasonic irrigation in conjunction with antiseptic agents yielded conflicting results from *in vitro* microbiological studies, although it generally would be more effective than needle-and-syringe irrigation in removing soft and hard tissue debris. All studies either examined the cleaning and disinfection effect of the ultrasonically activated irrigant [27], or looked into the file oscillation and/or flow of irrigant inside the canal [25,28,29,30]. Little information about the spatial cleaning ability due to the mechanical action or the biophysical effect of the endosonic file, especially in relation to those file locations with different vibration amplitudes. Thus, the aim of this study was to examine the spatial effect of ultrasonic irrigation on a mono-species biofilm in a simulated root canal model with an oblong cross section, correlated to the location of the nodes and anti-nodes along the endosonic file. The null hypothesis was that there is no difference in the cleaning effect of passive ultrasonic irrigation at various locations of the root canal.

## 2. Materials and Methods 

**Figure 1:**
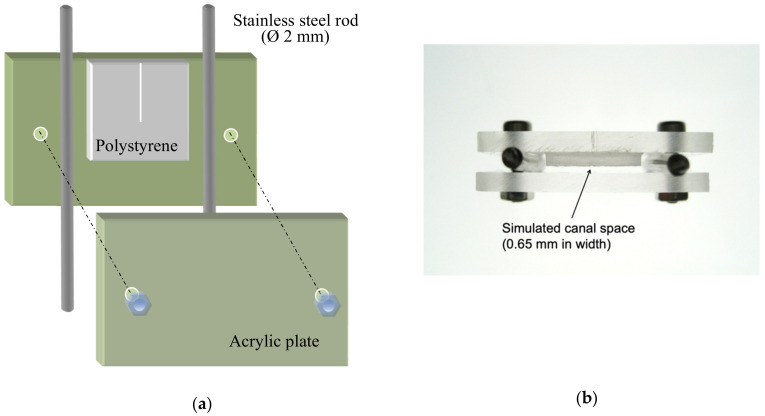
Artificial model simulating a canal with oblong cross section, akin to a wide isthmus: (**a**) Diagrammatic representation of the setup; (**b**) Top view of the actual model.

An artificial model, in the form of a rectangular box (Figure 1A), was constructed to simulate an oblong canal. It comprised two transparent acrylic plates (15 mm × 25 mm) separated by two stainless steel rods, 2 mm in diameter and 20 mm in length, at each end. One of the acrylic plates carried a 10 mm × 10 mm mirror-smooth polystyrene sheet (1.35 mm thick) with a 6 mm-long reference line marked on the mid-line extending from one edge of the sheet (Figure 1A). This line corresponded to the position of the endosonic file was positioned and aligned to this line during the experimentation.

**Figure 2:**
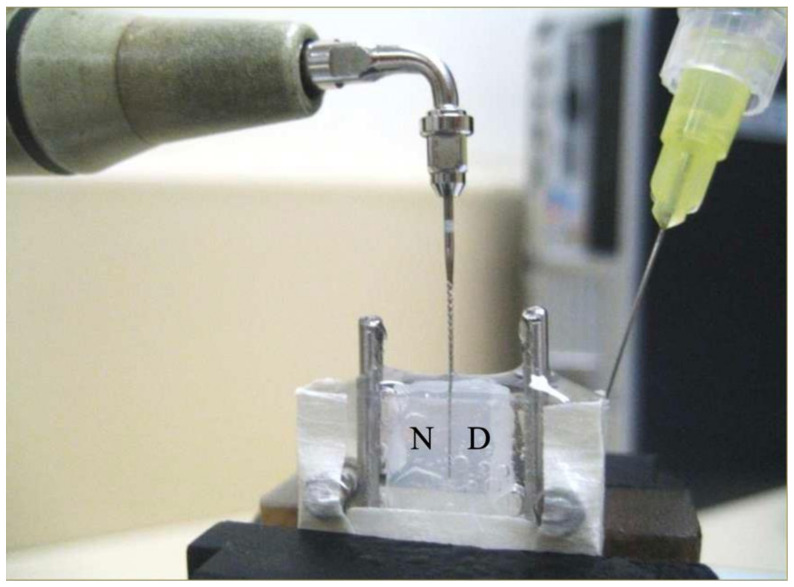
Experimental setup, with “N”= near-side to the ultrasonic handpiece, and “D”= distant side. Sterile saline solution was slowly injected into the simulated canal space to replenish any solution that was splashed out by the vibrating file.

A 7-day *Enterococcus faecalis* biofilm was cultivated on a polystyrene sheet before assembling the (pre-sterilized) canal model aseptically. Briefly, *E. faecalis* (strain ATCC 29212) was first inoculated on blood agar plate and cultured anaerobically for 24 h. One scoop of the bacteria was taken from a colony and transferred into sterile brain heart infusion (BHI) broth to make a suspension. The suspension was titrated to 1 × 10^7^ colony forming units (CFU)/mL (McFarland standard 4) by monitoring the optical density at 660 nm with a microtiter plate spectrophotometer (SpectraMAX 340 Tunable Microplate Reader; Molecular Devices, Sunnyvale, CA, USA). Aseptic technique was strictly followed. The acrylic plate carrying the polystyrene sheet was then placed in a culturing bottle with the polystyrene surface facing upward and exposing to the culture solution for bacterial colonization. The prepared *E. faecalis* suspension was added into the bottle until the entire specimen was covered. The bottle was put in an incubator-shaker (Orbital Incubator SI 500; Stuart, Staffordshire, UK) at 37 °C and 80 rpm for 1.5 h for bacterial adhesion. After that, the suspension/solution was drawn away completely from the bottom of the bottle by a pipettor without touching the biofilm surface. Then, 7 mL of sterile BHI medium was added, the bottle sealed with a lid and plastic cling-film (Parafilm “M”; Pechiney, Chicago, IL, USA), and incubated in the incubator-shaker at the same settings for a total of 7 days. The BHI medium was replaced daily during this period. After 7 days, the polystyrene plate was washed gently by waving it in 7 mL of PBS; the components of the canal model were fastened together with sterile stainless steel screws and nuts. The assembly left a space of (2.0 – 1.35 =) 0.65 mm width, between the acrylic plate on one side and the polystyrene sheet (with biofilm) on the other, to simulate an oblong root canal space, akin to a wide isthmus (Figure 1B). The bottom and the two edges of the box-shaped model were sealed with autoclave tape (3M, St. Paul, MN, USA) to prevent the irrigant from leaking out. The canal model was secured in a metal vice on an adjustable platform with the polystyrene sheet oriented vertically, guided by a plumb-line set. Sterile physiologic saline (Thai Otsuka Pharmaceutical, Samutsakorn, Thailand) was used as the irrigant in the experiment, to eliminate any confounding due to antimicrobial action of the solution. The saline was gently injected to fill up the space (Figure 2).

Prior to the experiment, a brand new, size 15 endosonic file (DT-007 Endosonores; Dentsply-Sirona, Ballaigues, Switzerland) was attached to an ultrasonic handpiece (Piezon Endo; EMS, Nyon, Switzerland), set to vibrate in a tank of water and was photographed. The positions of the nodes and anti-nodes for the vibrating file were measured in software for reference (Table 1). Then, another new endosonic file was mounted onto the ultrasonic handpiece with the file pointing vertically downward. The file was lowered into the middle of canal space to a depth of 6 mm, aligned with but not touching the reference line, nor any of the walls. The ultrasonic handpiece was oriented parallel to the polystyrene sheet, that is, along the lengthier axis of the rectangular cross section (Figure 1B).

**Figure 3:**
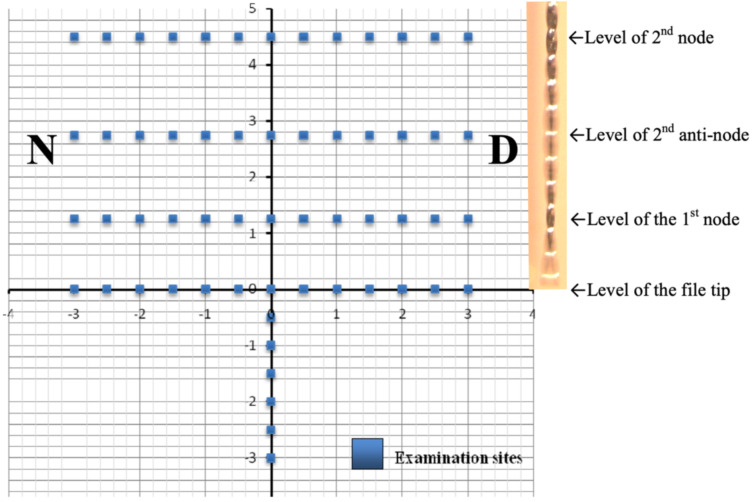
Schematic representation of the 58 examination sites, with the file aligned on the Y-axis and its tip situated at the origin. (Note: A photo of the vibrating endosonic file was inserted to illustrate the locations of the nodes and anti-nodes.)

The endosonic file was set to vibrate for 5 minutes, with sterile saline (overall volume used generally below 5 mL) being slowly injected into the space from the side to maintain the fluid level in the chamber (Figure 2). After the ultrasonification, the file was examined for any breakage. A total of four specimens were subjected to such passive ultrasonic irrigation, each with a new endosonic file. In addition, one negative (without biofilm inoculation) and one positive control (with biofilm but not ultrasonically irrigated) were included. After the experiment, the model was dissembled and the plate carrying the polystyrene (biofilm) sheet was gently waved in 7 mL of PBS, then subject to a LIVE/DEAD BacLight Bacterial Viability Kit (L7012 Invitrogen; Molecular Probes, Eugene, OR, USA) was used to quantify the amount of bacteria present. This kit comprises two nucleic acid-binding stains: SYTO^®^ 9 that makes viable bacteria fluoresce green; and propidium iodide that stains non-viable bacteria (with damaged cell membrane) red. After applying the dyes, the specimen was incubated at room temperature in the dark for 30 min and then examined under a confocal laser scanning microscope (CLSM) at ×400 magnification (IX81 FluoView FV1000; Olympus, Shinjuku-ku, Tokyo, Japan) at 488 and 543 nm. Digital images were acquired at 58 pre-determined areas throughout the polystyrene surface to visualize the spatial effect of that ultrasonic irrigation. Emphasis was placed on examining the biofilm situated near the nodes and anti-nodes of the vibrating file (Table 1), as well as areas apical to its tip (Figure 3).

Each specimen was divided into a “proximal” half that was situated close to the endosonic handpiece (denoted as the near-side, “N” in Figure 1B) and was assigned a negative value for the distance measurement on the x-axis (Figure 3). The distal half was denoted as “D” and assigned a positive value on the x-axis. The y-axis indicated the vertical position of the endosonic file. Six sites apical to the tip of the file were also examined for potential cleaning action beyond the file tip. 

All images were analyzed in software (Photoshop; Adobe, San Jose, CA, USA). The areas occupied by bacteria were selected automatically by thresholding in the software, with the tolerance setting adjusted individually to a level that incorporated all bacteria in each picture. The total number of pixels with bacteria was calculated as percentage of live (stained in green) and dead (red) bacteria, out of the overall size of the image (4,194,304 pixels). The evaluation was done by blinded, experienced technician, not involved in the design of the experiment, to minimize bias.

Depending on the normality of the data, statistical analyses were performed the Paired t-test, One-way Analysis of Variance (ANOVA) or the non-parametric Kruskal-Wallis Test in software (GraphPad; InStat, San Diego, CA, USA) at α = 0.05, After that, groups were compared by the parametric Tukey-Kramer Multiple Comparisons, or the non-parametric Dunn’s Multiple Comparison Test, where appropriate.

## 3. Results

### Quantification of Bacteria

**Figure 4:**
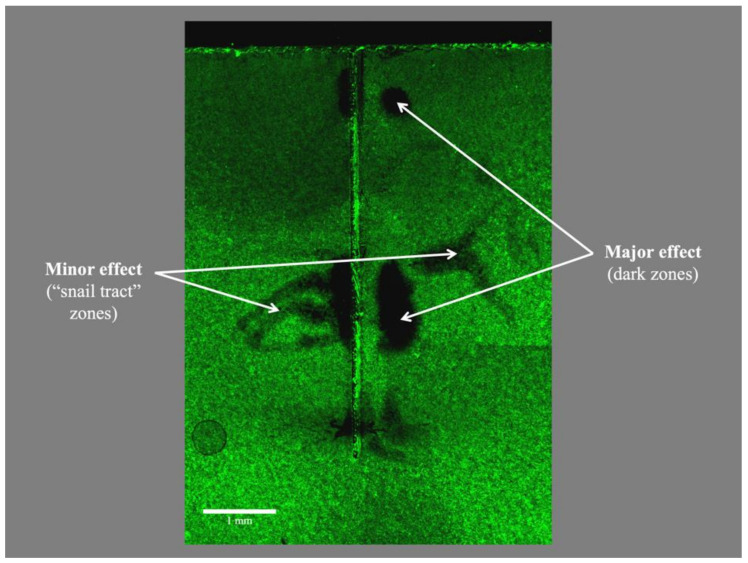
Typical appearance of the biofilm remaining on the polystyrene strip of the artificial canal model after passive ultrasonic irrigation, with viable bacteria stained and fluorescing in green. Notice (i) the absence of dead bacteria (that should have been stained in red), as sterile saline was used as the irrigant; (ii) non-uniform dislodgement of viable bacteria by the vibrating ultrasonic file which was held close to, but not in contact with that surface; and (iii) areas with dislodged bacteria were situated at a level approximating to the location of the anti-nodes of the vibrating file.

None of the ultrasonic files fractured after 5 min of unconstrained vibration while immersed in the irrigant solution, i.e., the passive ultrasonic irrigation (PUI) regime, inside the simulated canal model. Virtually no dead bacterial cells (stained red) could be identified; microorganisms remaining on the polystyrene plates were stained green i.e., viable (Figure 4). Minimal amount of area covered with viable bacteria was noted for the negative control (without biofilm cultivated), confirming sterility control of the setup.

**Figure 5:**
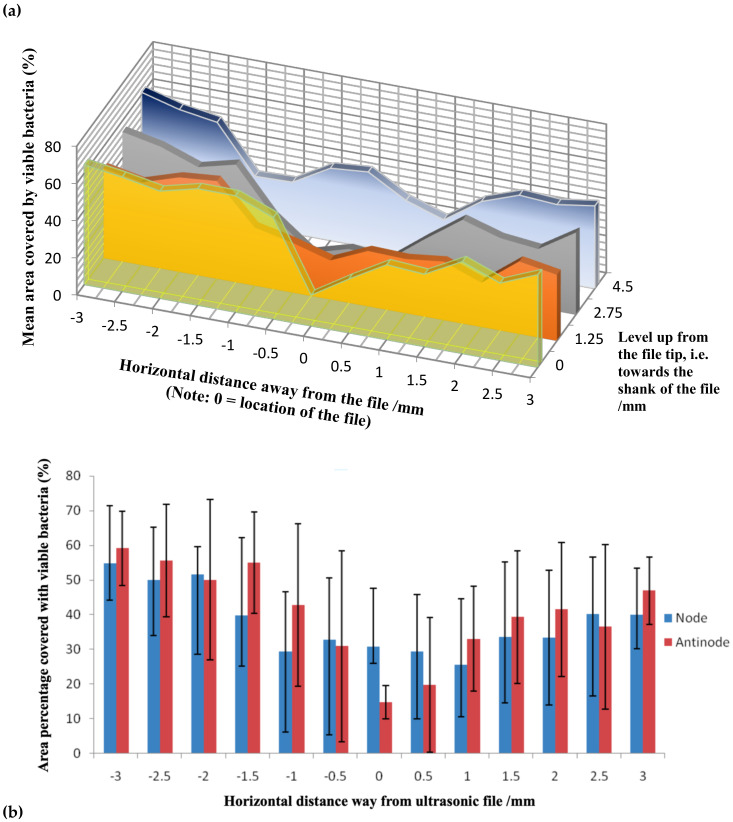
Mean area percentage covered by viable bacteria at various horizontal distances away the ultrasonic file: (**a**) Values at the four levels examined (0 = file tip where the first anti-node was situated) from the tip towards the shank of the file; (**b**) Mean values of viable bacteria for the collective levels of the nodes versus anti-nodes of the vibrating file, with 95% confidence intervals (approximately 2 S.D.) indicated by the whiskers.

For the experimental group, the lowest number of viable bacteria was generally observed over the reference line (the y-axis in Figure 3) where it was closest to the vibrating file, with the amount ranging from 13.1% (at the 2.75 mm-level coronal to file tip) to 40.5% (4.5 mm-level) (Figure 5). Relatively low amounts of viable bacteria were observed up to 1 mm away from the file (along y-axis), before the amount began to rise for sites farther away from the file. At 3 mm away, some 35.5 to 64.4% of the sampled area remained covered with viable bacteria, which value was significantly greater than that on the reference line (i.e., the y-axis where the file was aligned) or at 0.5 mm from the file (*P* < 0.01). Sites at the same level as the location an anti-node tended to be cleaner, compared with those corresponding sites at the level of a node. A significant difference in the amount of remaining bacteria was detected collectively between the level of nodes versus anti-nodes along the reference line (*P* = 0.050) along the file (Figure 5).

**Figure 6:**
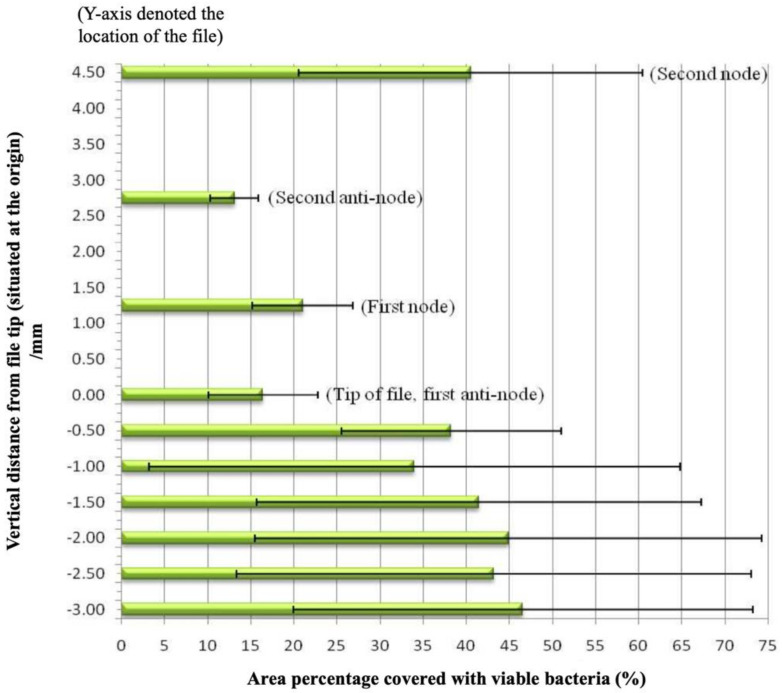
Mean area percentages (and 95% confidence interval) covered by viable bacteria at 4 levels along of the ultrasonic file, and beyond the file tip.

Apical to the vibrating file, the amount of bacteria was more than double at 0.5 mm away (38.2%) than that at the file tip (16.4%) (*P* < 0.05). Farther away, the value increased but tended to level off at about 45% (Figure 6). No significant difference was found between sites 0.5 mm and those that were farther away from the endosonic file tip (*P* > 0.05).

## 4. Discussion

*Passive Ultrasonic Irrigation* (PUI) may be defined as the ultrasonic activation of a file (or file-like insert) inside a pre-shaped root canal space filled with an aqueous irrigant (liquid medium), without touching the canal call, without any constraint or moving the file about. Knowing that endosonic file vibrates in a roughly elliptical manner along the director of the ultrasonic handpiece, when envelop of movement of the vibrating file is concerned [31], the oblong cross section was aligned with the major vibrating axis of the file to minimize any file contacts with the wall. We deliberately used a bland solution as the irrigant to examine the declumping or mechanical cleaning action of PUI on the *E. faecalis* biofilm. The endosonic file was set to be not touching the simulated canal wall during vibration, according to the definition of PUI, to avoid any scrapping away of the biofilm due to physical contacts. An SEM study has shown that PUI used in conjunction with 1% sodium hypochlorite was effective in eliminating *E. faecalis* biofilm from the root canal [22], but no detailed spatial distribution of the effect was described. In fact, as sodium hypochlorite was used in conjunction with the endosonic file in that study, it was difficult to quantify the biophysical effect attributable to the ultrasonic agitation alone. In the present study where physiologic saline was used, bacteria were dislodged from the biofilm rather than being killed, as was evidenced by the absence of dead microbial cells on the plate (see Figure 4). This indicated the lack of bactericidal action of ultrasound alone in the absence of an antiseptic or disinfecting agent.

It is a general belief that the cleaning action would be the most effective in areas situated in close proximity to the ultrasonic file (in non-contact mode). However, our results indicated that this statement is true only at the anti-nodes, but not for area near the location of the nodes where the amount of remaining viable bacteria was significantly greater than the former (Figure 5). As cavitation is unlikely to occur in the confines of a root canal [28], it is plausible for a greater cleaning effect to occur at regions where the file is vibrating more vigorously, i.e., at the anti-nodes, due to the rapid microstreaming of the solution that disrupted the biofilm from the polystyrene sheet in this study. As the file is “stationary” at the node, the cleaning effect would rely on secondary streaming of the irrigant at a slight distance away from the file [24]. Dentine substrate was not used to grow the biofilm in this study, which may be a limitation of the methodology. On the other hand, the present methodology was so designed to limit the amount of variables related to the use of biological tissues as the sample substrate. 

The greatest declumping or mechanical cleaning effect (13.1%) was observed at the 2.75 mm level that corresponded to the location of the second anti-node, instead of the file tip (16.4%) where the largest displacement amplitude is anticipated [25,26]. Our observation corroborated the findings of Felver et al. [29] who demonstrated by sonochemiluminescence that the tip of an ultrasonic scaler, despite showing the greatest vibration amplitude, does not exhibit spatially widespread effects. Taken together, those areas on the reference line (closest to the endosonic file) opposing the anti-nodes were significantly cleaner than at the nodes (Figure 5b). There was little difference elsewhere, regardless of their locations, although generally the farther (horizontal distance) away from the vibrating file, the lesser the cleaning effect could be found. This is expected, because the ultrasonic energy will dissipate while transmitting through the medium. The mechanical cleaning effect also dropped towards (i.e., moving up) the shank of the instrument, away from the file tip, after the second anti-node.

Krell and Johnson [32] have shown that the irrigant could penetrate to a depth of about 1 mm beyond the tip of a straight, diamond-coated, size 35 or 45 ultrasonic file. In contrary, using a much small file (size 15), our results indicated that the cleaning effect is minimal for areas beyond or apical to the file tip (Figure 6). Although the solution might have been circulated beyond the vibrating file tip, the streaming velocity of the irrigant appeared not to be sufficient enough to disrupt the biofilm there. Indeed, the cleaning effectiveness drops significantly even at a short distance (0.5 mm) beyond to the file tip. Thus, the (claim of any) cleaning ability beyond the tip of an endosonic file is unlikely to be the result of the ultrasonic activation, but more likely to be related to the disinfection ability of the irrigant, if that had been the case.

The creation of a standard root canal shape using natural teeth is hardly possible, because of the large variation in canal morphology [33]. As root canals are often oblonged in cross section, the dimension of the model in this study was so designed to allow the ultrasonic file to vibrate freely but without touching the (simulated) canal wall. Such mode of application was said to allow the best opportunity for microstreaming (and, possibly, cavitation when the most ideal condition could be achieved) to take place [30]. In the present experiment, to avoid any damping of the ultrasonic vibration and to reduce the chance for the vibrating file to hit the canal wall during vibration, the ultrasonic handpiece was so oriented that the vibrating file was in line with the long-axis of the oblonged cross section. A (polystyrene) flat surface was used to facilitate the CLSM examination, as that would minimize the background noise to enhance the accuracy of measurement. We recognized the possibility for the ultrasonic energy to bounce off a flat surface, as opposed to being scattered after hitting an irregular canal wall. However, that energy is likely to be either absorbed (and caused disruption of the biofilm) or dispersed in the fluid medium. In this study, the ultrasonic handpiece was kept stationary and one orientation of the handpiece was tested. Future investigation may be done to determine if the cleaning effect may differ when the ultrasonic handpiece is oriented in a different manner.

A single-species biofilm of *E. faecalis* was used in this study, as this microorganism has been implicated as an important inhabitant of endodontic failure cases in many studies [34,35,36]. Saline was used during ultrasonic irrigation, because this present report only aimed at studying the mechanical effect of ultrasonication. It has been shown that gram-positive anaerobes are less susceptible than gram-negative species to ultrasonication [37,38]. Hence, the ultrasonic irrigation was carried out for an extended period (five minutes) to visualize any impact of such mechanical action on this species. It is apparent from the present results that complete dislodgement of biofilm from a surface cannot be achieved by ultrasonic agitation of a bland solution alone. Given that PUI with the endosonic file being kept stationary at one position without touching the canal wall will not produce a uniform and consistent dislodgement of an adherent biofilm, it is recommended that the clinician should move the file about in the root canal space, even though it may touch and contact the canal wall, to encourage a more uniform removal of bacteria that may be attached there. Indeed, it has been shown that ensuring no contact with the canal wall during PUI is close to impossible to achieve, and that some contacts will occur during ultrasonic activation of the irrigant solution with an endosonic file [39]. Despite that fact, movement of the endosonic file during ultrasonic irrigation and the use of an antiseptic of disinfectant solution are recommended.

## 5. Conclusions

Passive ultrasonic irrigation, without any disinfecting agent, is able to remove a single-species *E. faecalis* biofilm only at sites that are situated in close vicinity of the endosonic file. The mechanical cleaning effect is neither uniform across the surface, nor along the length of the vibrating file. The ability of ultrasonic agitation to dislodge the bacterial biofilm beyond the tip of the endosonic file is limited. While ultrasonic activation of disinfectant solution is often practiced in clinical endodontic treatment, it is advisable not to hold the endosonic file stationary in one single position. 

## Figures and Tables

**Table 1:** Distance of the nodes and anti-nodes from the tip of a no. 15 endosonic file vibrating in water, and the locations where CLSM images were captured after the experiment.

	Distance From the File Tip/mm	Location on y-Axis (see Figure 2) Where Examination Was Made/mm
2nd Node	4.60	4.50
2nd Anti-node	2.71	2.75
1st Node	1.34	1.25
1st Anti-node	0	0
